# Surface bio-engineering of melt electrowritten tubular scaffolds via plasma immersion ion implantation (PIII)

**DOI:** 10.1016/j.mtbio.2025.101923

**Published:** 2025-05-29

**Authors:** Anyu Zhang, Anne Metje van Genderen, Bingyan Liu, Junyi Qian, Jirawat Iamsamang, Ziyu Wang, Miguel Castilho, Behnam Akhavan

**Affiliations:** a School of Biomedical Engineering, University of Sydney, Sydney, Australia; b School of Physics, University of Sydney, Sydney, Australia; c Sydney Nano Institute, University of Sydney, 2006, NSW, Australia; d Div. Pharmacology, Utrecht Institute for Pharmaceutical Sciences, Utrecht University, the Netherlands; e Department of Biomedical Engineering, Eindhoven University of Technology, Eindhoven, the Netherlands; f Institute for Complex Molecular Systems, Eindhoven University of Technology, Eindhoven, the Netherlands; g Charles Perkins Centre, University of Sydney, 2006, NSW, Australia; h School of Life and Environmental Sciences, University of Sydney, 2006, NSW, Australia; i School of Engineering, University of Newcastle, Callaghan, 2308, NSW, Australia; j Hunter Medical Research Institute (HMRI), Precision Medicine Program, New Lambton Heights, 2305, NSW, Australia

**Keywords:** Vascular tube, Biofunctionalization, Plasma surface engineering, Covalent immobilization

## Abstract

Melt electrowriting (MEW) enables the fabrication of highly controlled, open-pore tubular constructs for replicating the complex architectures of vascular, renal, and other tissues. However, a key challenge is to functionalize their surfaces so that they not only support but also instruct key biological interactions, particularly in promoting vascularization. Here, we propose plasma immersion ion implantation (PIII) as a biofunctionalization strategy for open-pore tubular constructs fabricated by MEW. Surface chemistry analysis confirmed homogeneous treatment across PIII-treated MEW 3D structures, while uniaxial tensile tests demonstrated no significant changes in mechanical properties following the treatment. Electron paramagnetic resonance (EPR) data provided evidence of the formation of a stable, radical-rich surface, which was further validated by fluorescence imaging with a model molecule, confirming the radicals’ role in enabling uniform covalent biomolecule attachment. The PIII-treated MEW constructs were covalently functionalized with vascular endothelial growth factor (VEGF), thereby modulating the behavior of seeded cells. Endothelialization studies using conditionally immortalized glomerular endothelial cells (ciGEnC) demonstrated that VEGF-immobilized MEW tubes effectively support monolayer formation, achieving outcomes comparable to those observed with VEGF supplementation in culture media. Remarkably, the immobilized VEGF sustained endothelialization with a similar effectiveness to traditional VEGF suspension methods over prolonged culture conditions (21 days), but without the need for continuous VEGF supplementation. These findings establish a novel biofunctionalization strategy for vascularized tissue engineering scaffolds and pave the way for plasma-modified MEW tubes as platforms for preclinical models and regenerative medicine applications.

## Graphical abstract

**Figure:**
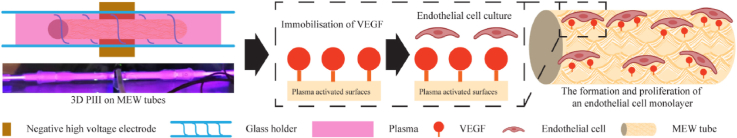

## 1 Introduction

Animal models have long been central to preclinical drug testing, yet their limitations in predicting human drug responses are becoming more evident with many drugs passing animal tests but failing in humans [1,2]. Beyond this, animal models are costly, low throughput, and raise ethical concerns [1,2]. These drawbacks have driven demand for more reliable, human-relevant models to assess drug efficacy and safety. Organ-on-a-Chip (OoC) systems, which are microfluidic devices to culture human cells, offer a promising solution to animal models by more accurately simulating human-specific (patho)physiology [2,3]. OoCs provide control over tissue microenvironments, allowing better *in vivo*-like conditions than traditional 2D *in vitro* models. These platforms have demonstrated significant potential for modeling various organs, including bone marrow [[4], [5], [6]], kidney [[7], [8], [9], [10], [11], [12], [13]] and lung [[14], [15], [16], [17], [18]]. A key aspect of many OoC systems is replicating tubular structures, which are fundamental building blocks for constructing more complex tissue and organ models [2,8,[19], [20], [21], [22]] containing vasculature, renal tubules, or the gastrointestinal tract [23]. In particular, the integration of vascular structures into such systems plays critical roles in nutrient delivery, waste removal, and maintaining homeostasis [1,21].

Previously developed OoC devices achieve simple vascularization by using perfusable platforms and porous scaffolds [[24], [25], [26], [27]], which fall short in replicating the complex function of blood vessels, especially the endothelial cell layers responsible for filtration and molecular trafficking [[28], [29], [30], [31]]. Therefore, creating vascularized porous tubular structures with endothelial cell layers is vital for advancing OoC technology [32].

To induce biomimetic vascularization in OoC systems, biofunctionalizing tubular porous scaffolds through the immobilization of pro-vascularization growth factors like vascular endothelial growth factor (VEGF) is a promising approach. This immobilization is normally achieved through traditional methods such as physical adsorption or wet-chemistry covalent immobilization techniques, such as EDC/NHS and polydopamine [[33], [34], [35]]. However, physical adsorption is limited by protein denaturation, desorption, and replacement [36,37], while covalent immobilization via wet-chemistry techniques requires complex processing and often leaves toxic residues [[33], [34], [35]].

Our research group has actively contributed to the development and application of plasma immersion ion implantation (PIII) for biofunctionalization of polymeric materials [35,38,39] to address the challenges outlined above. PIII functions by subjecting materials immersed in plasma to a negative potential bias to create energetic ion bombardment towards the surface [35,39,40]. In the case of polymeric surfaces, PIII can be applied to generate surface-embedded radicals [39]. The embedded radicals enable covalent attachment of diverse biomolecules, which process is versatile and simple, only requiring a one-step incubation of PIII-treated surfaces within biomolecule suspension [39,40]. As a result, PIII combines the simplicity of physical adsorption and the enduring stability of wet-chemistry covalent immobilization without inheriting their respective limitations.

Applying PIII to tubular scaffolds presents several challenges. PIII has primarily been used to bio-engineer 2D polymeric surfaces [39,41], as its energetic ion bombardment is a directional process that is difficult to apply uniformly on 3D structures. Recent studies have explored various plasma configurations to achieve homogeneous treatment on 3D materials [38,[42], [43], [44]]. However, the development of technology for uniformly treating additively manufactured open-pore, geometrically complex tubular microarchitectures—essential for supporting and guiding cells in native-like biological environments—remains limited.

An emerging high-resolution additive manufacturing technique to produce such biomimetic structures is melt electrowriting (MEW), which precisely deposits micro-sized fibers to create reproducible 3D porous, cell-instructive architectures [35,45,46]. Polycaprolactone (PCL), the current gold-standard polymer for MEW, has been treated with various surface coating methods including plasma methods [[47], [48], [49]]. However, PCL is highly heat-sensitive, and can be damaged by thermal accumulation during plasma processes [50]. PIII, in particular, produces energetic ion bombardment that induces minor temperature fluctuations, posing a risk of deforming delicate MEW-PCL structures and compromising their key properties, such as structural integrity and mechanical strength. Therefore, there is a need to advance PIII from 2D to 3D, while ensuring their compatibility with the open-pore, polymer-based tubular structures fabricated using MEW.

**Fig. 1:**
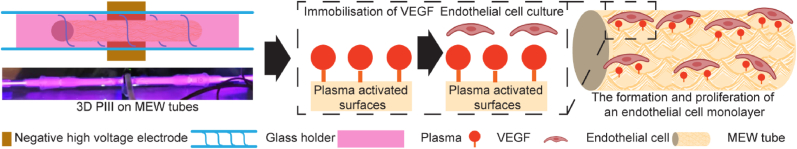
Schematic representation of the PIII biofunctionalization on 3D tubular porous structures. PIII enables the homogeneous covalent immobilization of VEGF that supports the formation and proliferation of an endothelial cell monolayer. The process flow summarizes the application of PIII on 3D porous tubes to produce biofunctionalized MEW tubes.

Here, we advanced PIII technology to engineer the 3D surfaces of tubular constructs designed to support and guide the formation of vasculature in, e.g., OoC devices. To achieve this, we first fabricated porous tubes with well-defined microarchitecture using MEW. We then investigated the biofunctionalization of these structures using the PIII technology, focusing on its ability to uniformly and covalently attach biomolecules (see Fig. 1). To evaluate the effectiveness of this approach, conditionally immortalized glomerular endothelial cells (ciGEnC) were cultured on VEGF-immobilized MEW tubes to assess their proliferation and maturation.

The findings presented in this study establish PIII as a versatile and enabling technology for homogeneous surface activation of MEW tubes while preserving their porous architecture. The treated tubes facilitated uniform covalent biomolecule immobilization and supported ciGEnC monolayer formation and proliferation, highlighting their potential as biomimetic architectures for diverse applications in tissue engineering, regenerative medicine, and biofabrication, including organ-on-chip (OoC) platforms and beyond.

## 2 Materials and methods

### 2.1 Melt electro writing (MEW)

Tubular scaffolds were printed using a custom-built MEW device consisting of a rotating aluminum mandrel (∅ = 1 mm) mounted on an x-y axis and a custom print head mounted on a movable z-axis. The x-y-z axes were computer-controlled using G-code and commercially available software (Motion Perfect V4.2, Trio Motion Technology Ltd., Gloucestershire, UK). Mandrel rotation was controlled by Arduino IDE software, and all movement was executed using an advanced MC403 motion controller (Trio Motion Technology Ltd.). Medical grade polycaprolactone granules (PCL, Purasorb PC12, Corbion, Gorinchem, The Netherlands; Molecular weight of 72 kDa [51]) were loaded into a glass syringe with a 27G size metallic nozzle and heated to 88 °C for 30 min. A high-resolution air pressure regulator (VPPE-3-1-1/8-2-010-E1, Festo, Delft, The Netherlands), and a high voltage source (Heinzinger, LNC 30000-5 POS, 0–30 kV, Rosenheim, Germany), kept constant at 2.5 bar and 6.2 kV, respectively, extruded and electrified the PCL into thin fibers. The distance between the nozzle and the mandrel was maintained at 4 mm. Printed PCL MEW tubules were designed using an open-source software [52], and had 8 pivot points and 20 layers stacked on top of each other, with a pore size of 0.53 mm^2^, length of 22.45 mm, and a winding angle of 30°. The effective printing speed (Ve_ff_), a vector obtained by combining translational and rotational velocity, was 3 mm/s.

### 2.2 Plasma immersion ion implantation (PIII)

**Fig. 2:**
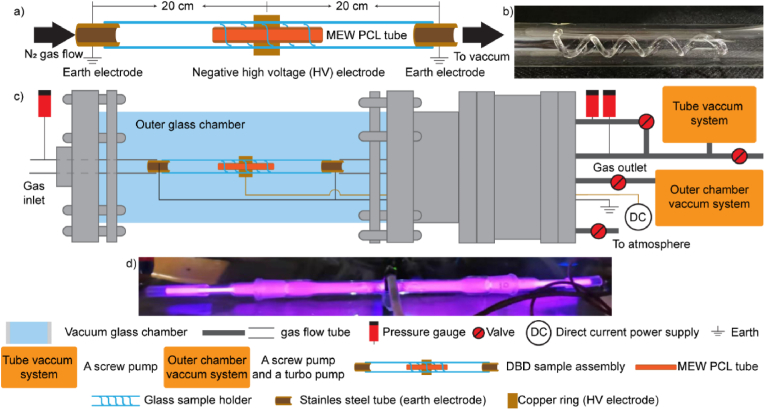
**a)** Schematic representation of the DBD sample assembly configured for surface activating 3D porous MEW PCL tubes. The MEW PCL tubes, placed in a glass holder, and the negative high-voltage (HV) electrode were centrally positioned between two grounded electrodes, which were symmetrically placed 20 cm from the HV electrode. **b)** Glass holder with a spiral inside. **c)** Schematic representation of the customized PIII reactor. The DBD sample assembly was isolated from the outer vacuum glass chamber. **d)** Optical image showing plasma discharge throughout the DBD assembly.

The PIII surface treatment of the 3D porous MEW tubes was executed using a customized plasma reactor designed with dual vacuum lines and a pulsed voltage source (RUP 6–25, GBS Electronics, Germany). Pressure gauges were placed to monitor gas flow pressure at the inlet and outlet of a dielectric barrier discharge (DBD) sample assembly, as well as inside the vacuum glass chamber, as depicted in Fig. 2. The MEW tube was positioned in a custom-made glass holder with a spiral structure inside (Fig. 2 b). Central to the setup was a copper ring electrode (length: 1.5 cm; inner diameter: 13 mm) wrapped around the outside of the glass holder. The copper ring electrode, connected to the pulsed voltage source, served as a negative high voltage (HV) electrode. Two earth electrodes were placed 20 cm away from the center of the copper electrode to complete the electrical configuration.

After the sample placement inside the glass chamber, the outer glass chamber was evacuated to a pressure of below 5 × 10^−3^ Torr using both turbo and screw pumps to prevent potential electrical arcing. The DBD sample assembly was evacuated to a base pressure of below 1.0 × 10^−2^ Torr using screw pumps. Subsequently, nitrogen (N_2_) gas was introduced into the DBD sample assembly through a flow controller (Alicat Scientific, USA), with pressure manually tuned via valves. Nitrogen was supplied at a flow rate of 1 standard cubic centimeter per minute (sccm), maintaining an internal pressure of 1 Torr. The PIII treatments were conducted at a bias voltage of −6 kV (pulse duration: 20 μs, frequency: 1 kHz) applied to the HV copper electrode. The influence of treatment time on PIII-treated 3D porous MEW tubes was studied by assessing MEW tubes treated for 5, 10, 20, and 60 min.

### 2.3 X-ray photoelectron spectroscopy (XPS)

The surface chemistry of untreated and PIII-treated MEW tubes, subjected to various treatment times, was examined using XPS on a K-Alpha + system (Thermo Scientific America). Survey spectra were obtained with a 200 μm measurement spot size, using 10 scans within the 0–1350 eV range, a pass energy of 200 eV, and a resolution of 1.0 eV. High-resolution (high-res) spectra were recorded at the same spot with 10 scans, a pass energy of 50 eV, and a resolution of 0.1 eV. All measurements were taken from samples aged one week after PIII treatment. To evaluate the homogeneity of the PIII treatment along the MEW tubes, XPS analysis was performed at the upstream, middle, and downstream sections, referred to as inlet, middle, and outlet, respectively. The XPS data were analyzed using Avantage software, with the peaks of C1s high-res spectra fitted to equal values of full width at half maximum (FWHM).

### 2.4 Attenuated total reflectance Fourier transform infrared spectroscopy (ATR-FTIR)

The chemical composition of the MEW tube surfaces was analyzed using a Bruker Vertex 80v spectrometer (PIKE, USA). The spectrometer was equipped with a germanium crystal plate (2 mm, PIKE, USA). The MEW tube was affixed onto an indium tape and placed under the full coverage of the crystal. Measurements were conducted under a vacuum of 2.5 hPa. Each sample was tested across a wavelength range of 4000 to 400 cm^−1^, with a resolution of 4 cm^−1^, and 32 scans. To evaluate the homogeneity of the PIII treatment along the MEW tubes, FTIR analysis was also performed at the inlet, middle and outlet sections of the MEW tubes.

### 2.5 Scanning electron microscopy (SEM)

A Phenom XL Scanning Electron Microscope (PhenomWorld, Eindhoven, The Netherlands) equipped with a secondary electron detector (SED) was employed to capture images of untreated and PIII-treated MEW tubes. The imaging process was carried out using the following parameters: a voltage of 10 kV, a pressure of 1 Pa, and a working distance of 5 mm in SEM mode. Prior to the SEM imaging, the MEW tubes were initially coated with a 16 nm-thick layer of Au/Pd using a sputter coater (SC7620 Quorum).

### 2.6 Electron paramagnetic resonance spectroscopy (EPR)

The quantification of free radical concentrations on the PIII-treated MEW tubes was conducted using a SpinscanX EPR spectrometer (Adani, Belarus). For the analysis, the MEW tubes were securely affixed to the bottom of an EPR quartz tube, ensuring their stable positioning at the center of the EPR chamber. The EPR analysis was performed with a microwave frequency set at 9.8 GHz and a magnetic power of 25 mW. The magnetic field was centered at 336 mT, with a modulation amplitude of 3 G. Ten scans were acquired for each sample, and the averaged spectrum was reported.

### 2.7 Mechanical evaluation

Mechanical characterization was performed with a tensile machine (CellScale® Biotester 5000, Canada). PIII-treated MEW tubes (PIII-20) and untreated tubes (UT) were sutured to the M1 bolts, embedded in the custom-made grips (Fig. 5a). Tensile testing was performed from an initial length (L_0_) of 4–5 mm, defined as the edge-to-edge distance between two grips (L_0_), at a strain rate of 100 % per minute. The engineering stress (σ) and strain (*ε*) were calculated using Equations (1), (2)), respectively, where F represents the tensile force registered by a 2.5 N load cell (sampling rate 1 Hz), r is the radius of the tube (0.5 mm), t is the average thickness (N = 5) measured using a digital microscope (Keyence VHX-970FN), L is the distance of the grips reported by the tensile machine, and, therefore, the specimen's displacement is L - L_0_. The yield point was defined by a maximum *ε* within the linear part of the elastic region before onset of plastic deformation, determined by least-squares regression (R2 > 0.98). The elastic modulus (E) was derived from the regression slope, while the strain energy density (U_0_) was calculated from the area under the σ-ε curve from *ε* = 0 to the yield point (Fig. 5b).

(1) $$\sigma =\frac{F}{\pi {[\left(r+t\right)}^{2}-r^{2}]}$$

(2) $$\varepsilon =\frac{L}{L_{0}}-1$$

### 2.8 Wettability analysis

Wettability measurements of the untreated and PIII-treated MEW tubes, standardized by weight and geometry, were conducted using a K100 tensiometer (Kruss Scientific, Germany). During each test, the MEW tubes were automatically driven to approach the liquid-air interface, initiating liquid absorption. Based on the capillary effect, the K100 tensimeter measures the mass change of adsorbed liquid as a function of time after the MEW tubes contact the liquid. The absorption behaviors of untreated and PIII-treated tubes were compared using MilliQ water as the testing liquid. The sorption characteristics of the PIII-treated tubes were further evaluated using n-hexane (anhydrous, 95 %, Sigma-Aldrich, USA).

A stainless-steel single fiber sample holder (model SH0801 for K100, Kruss Scientific, Germany) was used to fix the MEW tubes. The MEW tubes were aged for one week after PIII treatment, and then were cut into halves, with one end affixed vertically on the SH0801 sample holder using carbon tape. This setup facilitated accurate and reproducible wettability measurements, aiming to reflect weight changes over time during the capillary absorption. All measurements were carried out in a controlled environment in a fume hood.

### 2.9 Fluorescence visualization of covalent attachment

The covalent attachment and uniform distribution of biomolecules on PIII-treated MEW tubes was visualized using Cyanine5-labeled goat anti-rabbit IgG protein (Cy5-IgG, Invitrogen) as a model molecule. Both untreated and PIII-treated MEW tubes were incubated in a 4 μg/mL Cy5-IgG in phosphate-buffered saline (PBS) solution at room temperature for 2 h to allow covalent immobilization. Following incubation, the Cy5-IgG-coated samples underwent a detergent wash 4 times with 2 % (v/v) Tween-20 at 60 °C for 15 min each to remove any physisorbed IgG molecules and ensure that only covalently bound IgG molecules were analyzed. Subsequent rinses were performed three times with PBS to remove any residual detergent. For visualization, the tubes were imaged under a confocal microscope (Nikon C2, Nikon Corporation, Japan). The microscope settings were adjusted to a laser wavelength of 640 nm and a laser power setting of 2.0, optimized and kept consistent for detecting the Cy5 fluorescence. Image processing and analysis were conducted using Fiji software, with all post-processing, including contrast and brightness adjustments, uniformly applied across all images.

### 2.10 Cell proliferation

Conditionally immortalized glomerular endothelial cells (ciGEnC) were kindly provided by the group of Prof. J. van der Vlag (RadboudUMC, Nijmegen, The Netherlands) via an MTA with Prof. S. Satchell (Bristol Medical School (THS), Bristol, UK) [53]. CiGEnC proliferate at the permissive temperature of 33 °C and maturate at 37 °C. The cells were cultured in T75 flasks (Greiner Bio-One, Alphen aan den Rijn, The Netherlands) in Endothelial Cell Basal Medium-2 (Lonza, Basel, Switzerland) containing Microvascular Endothelial Cell Growth Medium-2 SingleQuots Kit (EGM-2 MV, Lonza, Basel, Switzerland).

### 2.11 Covalent protein immobilization

Following APPJ functionalization of MEW tubular scaffolds, the scaffolds were sterilized by washing the scaffolds 3 × 5 min, with 5 % (v/v) Penicillin-Streptomycin (Gibco, 15140122) in phosphate buffered saline (PBS, Lonza) and exposing them to UV-light (within a laminar flowhood) for 30 min. After sterilization, scaffolds were submerged in VEGF (Miltinyi Biotec, 130-109-384) in PBS solutions with different concentrations (500 or 1000 ng ml^−1^) for 24h at 4 °C.

### 2.12 CiGEnC seeding and expansion on MEW tubular scaffolds

Before seeding, the following tubular scaffold groups were prepared: a) PIII treated, and submerged in high VEGF concentration (1000 ng/ml, +PIII + VEGF high), b) PIII treated, and submerged in low VEGF concentration (500 ng/ml, +PIII + VEGF Low), c) PIII treated, not submerged in VEGF and no VEGF used in the medium (+PIII -VEGF), d) non-treated, not submerged in VEGF, but cultured in Endothelial Cell Basal Medium-2 containing Microvascular Endothelial Cell Growth Medium-2 SingleQuots Kit (VEGF containing). CiGENCs were seeded inside all tubular scaffolds at a concentration of 15 million cells/ml, using a volume of 50 μl. Tubular scaffolds were turned every hour, and after 4 h, submerged in medium. All PIII-treated groups were cultured in a non-VEGF medium; the non-PIII-treated group was cultured in a VEGF-containing medium (control). Scaffolds with cells were put at 33 °C and monitored for monolayer formation using brightfield microscopy. After obtaining a monolayer (within 1–3 weeks), scaffolds were transferred to a 37 °C incubator and cultured for an additional week, whereafter scaffolds were fixed for immunofluorescent staining.

### 2.13 Immunofluorescent staining

Following maturation, MEW tubes were fixed with paraformaldehyde (4 % v/v in PBS (Pierce™, Thermo Fisher Scientific, Carlsbad, CA, USA), 10 min) at room temperature (RT), followed by washing with 0.1 % PBS-Tween20 (v/v Sigma-Aldrich, Saint Louis, MO, USA). Later, the constructs were treated with a permeabilization buffer (0.3 % Triton X-100 (Sigma-Aldrich, Saint Louis, MO, USA)) for 10 min. After washing, the blocking solution (2 % FBS v/v, 0.1 % Tween-20 v/v, 0.5 % bovine serum albumin (w/v) in PBS was added for 30 min at RT. For immunofluorescence analysis, the primary (mouse anti-CD31 1:150 (Ab119339, abcam), goat anti-collagen IV 1:50 (1340-01, Southern Biotech) and secondary antibodies (Phalloidin Alexafluor 594 1:1000, AlexaFluor 488 Donkey anti Mouse 1:200, AlexaFluor 647 Doneky anti Goat 1:200, Invitrogen) were diluted in blocking buffer prior to application; primary antibodies were incubated for 90 min, while secondary antibodies were incubated for 60 min, both at RT. Thereafter, samples were incubated with DAPI for 8 min at RT. A Leica TCS SP8 X confocal microscope (Leica, Wetzlar, Germany) and Leica Application Suite X software were used to image immunofluorescent PCL MEW tubules. ImageJ (National Institutes of Health, Bethesda, MD, USA) free software was used to make Z-projections and to quantify cell alignment via actin filament direction with the directionality function and Fourier components analysis.

### 2.14 Statistical analysis

Data were presented as the mean ± standard error with a minimum of three replicates (n ≥ 3). Statistical differences were assessed using either one-way or two-way ANOVA. Statistical significance was indicated as ∗, p < 0.05; ∗∗, p < 0.01; and ∗∗∗, p < 0.001.

## 3 Results and discussion

### 3.1 Optimizing PIII treatment time for MEW tube surface activation

The development of bioinstructive MEW tubes requires stable and uniform immobilization of biomolecules on their porous tubular structure. A crucial step in preparing the biomolecule immobilization involves applying PIII to the tube surfaces to introduce radical-containing chemical structures that can form covalent bonds with the biomolecules on contact. A customized PIII configuration was developed and used for homogeneous surface treatment of the MEW porous tubular structures. The PIII system employed a spiral glass holder to position the MEW tubes, allowing plasma species to diffuse through the open porous structure and ensure exposure of all surfaces to the plasma for homogenous treatment (Fig. 2). In this section, we characterize and provide an understanding of the surface physicochemical changes in MEW tubes as a function of PIII treatment time.

**Fig. 3:**
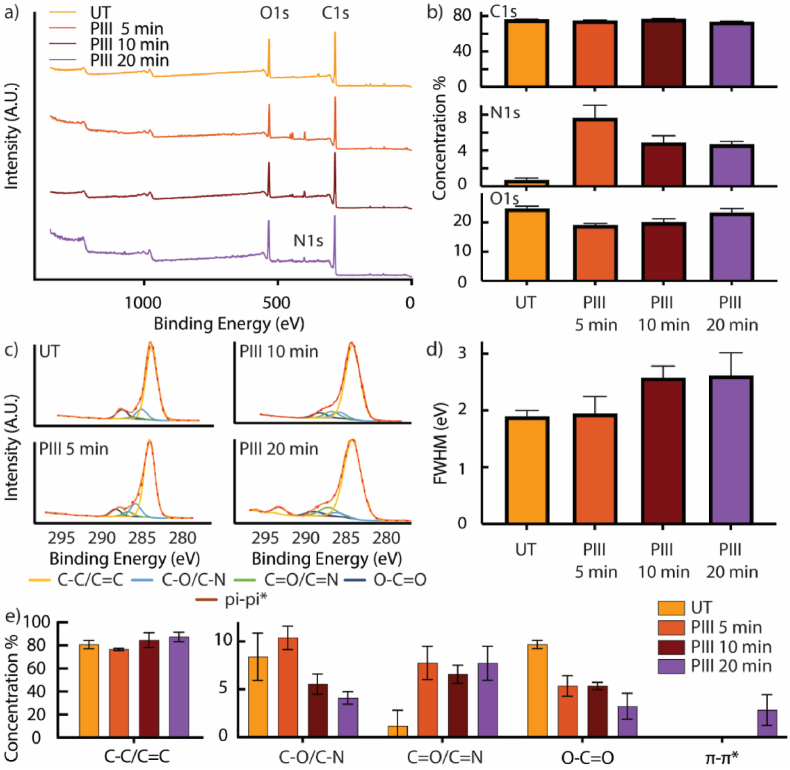
Effect of PIII treatment on surface chemistry of PCL MEW tubes. **a)** XPS survey spectra for the untreated MEW tubes and the MEW tubes treated after 5, 10, and 20 min. **b)** The calculated atomic concentration of C1s, N1s, and O1s for the untreated MEW tubes and the MEW tubes treated after 5, 10, and 20 min. **c)** XPS high-res spectra for the untreated MEW tubes and the MEW tubes treated after 5, 10, 20 min, and their peak-fitting using C1: C–C at binding energy (BE) ≅ 284.8 eV, C2: C–O/C-N at BE ≅ 286.7 eV, C3: C=O/C=N at BE ≅ 287.4 eV, C4: O-C=O at BE ≅ 289.0 eV, C5: π-π∗ at BE ≅ 293.4 eV. **d)** The Full Width at Half Maximum (FWHM) of the fitted chemical components for the untreated MEW tubes and the MEW tubes treated after 5, 10, and 20 min. **e)** The elemental concentration of the fitted chemical components.

The surface chemistry changes on the MEW tubes induced by PIII treatment were examined using XPS. Fig. 3a shows the XPS survey spectra for samples treated for 5, 10, and 20 min (PIII-5, PIII-10, PIII-20), with the atomic concentrations of C1s, N1s, and O1s, calculated from the survey spectra, plotted in Fig. 3b. The XPS survey spectrum for the untreated PCL control showed photoelectron signals from carbon and oxygen atoms, consistent with the PCL chemistry, (C_6_H_10_O_2_)_n_. Following the PIII treatment, a nitrogen peak was observed regardless of the treatment time (Fig. 3a), with the atomic concentration of N1s significantly increasing in PIII-treated samples (4.56 % ± 0.468 %–7.51 % ± 1.55 %) compared to the untreated control (0 %) (Fig. 3b). As nitrogen originated solely from the nitrogen plasma and was absent in untreated PCL, the occurrence of the nitrogen peak indicates successful nitrogen implantation into the PCL surfaces in all groups.

The influence of treatment time on creating PIII-treated surfaces on MEW tubes demonstrated a varying trend on different chemical elements. Initially, a decrease in the O1s concentration was observed in the PIII-5 group compared to the untreated samples. The initial reduction of relative O1s concentration could be associated with the introduction of nitrogen from nitrogen ion implantation onto the surface. As the treatment time increased, O1s concentration increased from 18.71 % ± 0.94 % in the PIII-5 group to 22.97 % ± 1.78 % in the PIII-20 group. This increase was accompanied by a decrease in N1s concentration from 7.51 % ± 1.55 % in the PIII-5 group to 4.56 % ± 0.468 % in the PIII-20 group (Fig. 3b). During PIII, the nitrogen ion implantation facilitates the breakdown and reorganization of carbon-carbon chains, resulting in carbonized structures containing embedded radicals [39,41]. Once the PIII-treated polymer is removed from the reaction chamber, the embedded radicals interact with atmospheric oxygen, resulting in surface oxidation [54]. Therefore, the increase in O1s concentration indicates increased surface oxidation, suggesting that the PIII-treated surfaces become increasingly active with increasing treatment time. The surface oxidation also explains the reduced N1s atomic concentration, as more oxygen elements were introduced to the surfaces.

Further assessment of the PIII treatment on the MEW tubes as a function of treatment time was performed using C1s high-resolution XPS spectra. The C1s chemical components (C1: C–C at binding energy (BE) ≅ 284.8 eV, C2: C–O/C-N at BE ≅ 286.7 eV, C3: C=O/C=N at BE ≅ 287.4 eV, C4: O-C=O at BE ≅ 289.0 eV, C5: π-π∗ at BE ≅ 293.4 eV) [55,56]. were fitted into the C1s high resolution spectra (Fig. 3c). The spectrum of untreated PCL displayed only C1, C2, and C4 Peaks, consistent with the PCL chemical structure, which consists of C–C, C–O, and O-C=O functional groups. After PIII treatment, the C3 peaks occurred, assigned to C=O/C=N, suggesting successful nitrogen ion implantation and surface oxidation on the PIII-treated surfaces. This observation aligns with a significant increase in the Full Width at Half Maximum (FWHM) of the C1s chemical components from 1.90 ± 0.11 eV in untreated samples to 2.61 ± 0.41 eV in PIII-20 groups (Fig. 3d). Such an increase indicates the creation of a more complex chemical environment around the carbon atoms after PIII treatment due to nitrogen ion implantation and surface oxidation.

The surface chemistry change was further analyzed by quantifying the area percentages of the components fitted under the C1s high-resolution spectra (Fig. 3e). The C3 peak showed a significant increase in the calculated concentration from 1.17 % ± 1.56 % in the untreated control to 7.73 % ± 1.78 % in the PIII-20 group, indicating increased nitrogen ion implantation and surface oxidation. This increase was accompanied by a decrease in the C2 peak concentration from 10.4 % ± 1.22 % in PIII-5 group to 4.10 % ± 0.65 % in the PIII-20 group, and a decrease in the C4 peak concentration from 9.69 % ± 0.44 % in the untreated control to 3.23 % ± 1.36 % in the PIII-20 group. Such changes in C1s chemical components suggest that the source of the surface oxidation was the increase of the peak, assigned to C=O/C=N. Moreover, as the N1s atomic concentration decreased (Fig. 3b), the primary source of surface oxidation was identified as the increase in C=O groups.

For the PIII-20 samples, a shift up (C5: π-π∗) at BE of 293.4 eV was observed with a concentration of 2.8 % ± 1.61 %, positioned 8.6 eV away from the main C1 peak (Fig. 3c–e). The occurrence of π-π∗ peak was attributed to extended delocalized electrons in sp2 hybridized carbons, often found in carbonized structures such as aromatic rings and ring-like chemical structures [34,35,39]. The formation of these carbonized structures can be explained by the destruction and reconstruction process of C1s chemical components. During the PIII treatment of PCL, O-C=O and C-O groups were destructed, as evidenced by the decrease of C2 and C4 peak concentrations (Fig. 3e). This observation is consistent with a previous study demonstrating that PIII leads to the destruction of C-C, O-C=O, and C-O functional groups in PCL films [57]. Such destruction is facilitated by the energetic nitrogen ion implantation and leads to the reconstruction of C=C and π-π∗ conjugated structures (Fig. 3e). As the treatment time increased, the destruction and reconstruction process became more evident, eventually resulting in a densely carbonized layer detectable by XPS (Fig. 3e) [57]. The carbonized layer serves as a reservoir for surface-embedded free radicals, characterized by the delocalized electrons in sp2-hybridized carbons. The presence of the surface-embedded free radicals has been previously demonstrated as a crucial feature, enabling reagent-free covalent immobilization of biomolecules on PIII-treated polymers [34,35,39]. The presence of the π-π∗ peak in only the PIII-20 group indicates that the successful surface activation of the PCL MEW tubes was achieved after 20-min PIII treatment.

### 3.2 Achieving homogeneous PIII treatment of MEW tubes

**Fig. 4:**
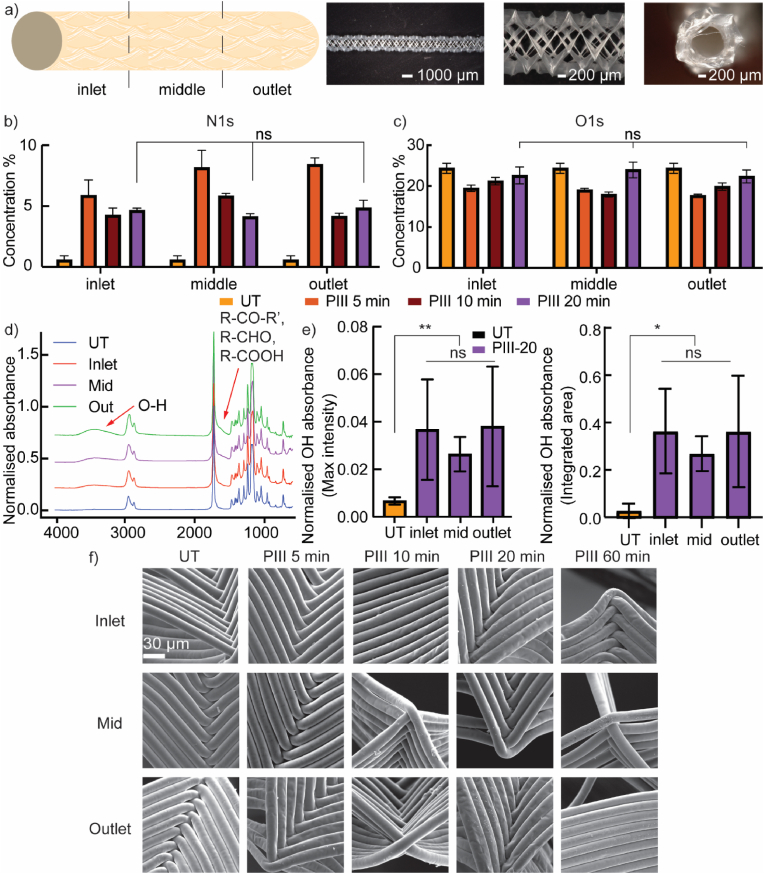
Evaluation of PIII treatment homogeneity on surface chemistry and topography on MEW tubes. **a)** Stereoscopic representation and optical images (from 20 × to 100 × ) of the MEW tube. The characterized upstream, middle, and downstream sections were referred to as inlet, middle, and outlet, respectively. The calculated atomic concentration of **b)** N1s and **c)** O1s distributed along the longitudinal cross-section of the untreated MEW tubes and the MEW tubes treated after 5, 10, and 20 min. **d)** FTIR spectra distributed along the longitudinal cross-section of the MEW tubes treated after 20 min, compared to the untreated MEW tubes. **e)** The normalized OH absorbance, both max intensity and integrated area, distributed along the longitudinal cross-section of the MEW tubes treated after 20 min, compared to the untreated MEW tubes. **f)** The SEM images are distributed along the longitudinal cross-section of the untreated MEW tubes and the MEW tubes treated after 5, 10, and 20 min.

Achieving homogeneous PIII treatment on 3D porous MEW tubes is needed for the uniform covalent immobilization of biomolecules. The homogeneity of the PIII treatment was evaluated by spatially mapping the surface chemistry distribution along the longitudinal cross-section of the PIII-treated MEW tubes, specifically measuring N1s and O1s atomic concentrations using XPS (Fig. 4a–c). Given the open-pore architecture of the MEW tubes, with fibers aligned perpendicular to the circumference (Fig. 4a), all surfaces were accessible and contributed to the surface chemistry measurements. The PIII-5 and PIII-10 groups exhibited variations in N1s atomic concentration along their longitudinal cross-sections, indicating inhomogeneity in the PIII treatment (Fig. 4a). In contrast, measurements from the PIII-20 group showed no significant differences in N1s atomic concentration at all positions (Fig. 4b), indicating that homogeneous nitrogen implantation was achieved after 20 min of PIII treatment. Similarly, the O1s results showed no significant difference in O1s atomic concentration at all positions in the PIII-20 group (Fig. 4c). As discussed in Section 3.1, the change of N1s and O1s concentration indicated nitrogen implantation and surface oxidation, which correlates with the formation of the carbonized layer. Therefore, the observed homogeneous distribution of both N1s and O1s confirmed a uniformly carbonized structure achieved after 20 min of PIII treatment. This finding also aligns with the observation that carbonized structures were only observed in the PIII-20 group. Given that biomolecule immobilization occurs at the outermost surfaces of the PIII-treated carbonized layer [54], the homogeneous PIII treatment provides a solid foundation for the uniform covalent immobilization of biomolecules on MEW tubes.

The homogeneous surface treatment was also validated by ATR-FTIR measurements taken at the same spatial distribution of the measurement points. The ATR-FTIR spectra exhibited a broadened peak in the region of 3100–3700 cm^−1^, attributed to the hydroxyl (O–H) stretching vibration band, and an increase at the lower wavenumber side of the ester carbonyl stretching (O–C=O) peak at 1726 cm^−1^ (Fig. 4d), which is assigned to ketones, aldehydes, and carboxyl groups. These functional groups contain oxygen, confirming the surface oxidation on PIII-treated surfaces observed in the XPS analysis. The absorbance of hydroxyl groups was quantified to assess the homogeneity of the PIII treatment on MEW tubes. There were no significant differences in absorbance across all positions (Fig. 4e), further validating the homogeneous surface chemistry distribution in the PIII-20 group.

Despite the effectiveness of PIII treatment, concerns exist regarding its potential impact on the structural integrity of MEW tubes. PCL, commonly used for melt electrowriting, has a low melting point of up to 60 °C and a glass-transition temperature (*T*_g_) of up to −60 °C [58]. This concern arises because PIII treatment involves energetic ion bombardment, which can generate heat in the PCL, while the PIII process occurs in a vacuum, an environment not conducive to heat dissipation. The influence of PIII on the structural integrity of MEW tubes was evaluated by assessing SEM images of MEW tubes treated as a function of treatment time. As shown in Fig. 4f, no structural melting occurred along the length of the MEW tubes for all sample groups, even with PIII treatment times extended to 60 min. This observation on the surface topography suggests that the PIII settings used in this study do not compromise the structural integrity of the MEW tubes.

Based on the surface chemistry and topography analysis data, we conclude that effective and homogeneous PIII treatment of the 3D porous MEW tubes was achieved with a treatment time of 20 min. This treatment time, along with the experimental settings of 1 Torr pressure and −6 kV voltage, was subsequently applied as the optimal setting for the following experiments.

### 3.3 PIII treatment maintains the mechanical properties of MEW tubes

Mechanical resilience is an important requirement for vascularized MEW scaffold constructs intended for dynamic tissue environments. A distinctive advantage of MEW technology lies in its ability to precisely tailor scaffold microarchitecture, enabling control over mechanical behavior even when using stiff thermoplastics. For example, previous studies have demonstrated that hexagonally microstructural scaffolds fabricated from stiff thermoplastic PCL, a polymer with limited deformability, can withstand dynamic loading conditions [59].

**Fig. 5:**
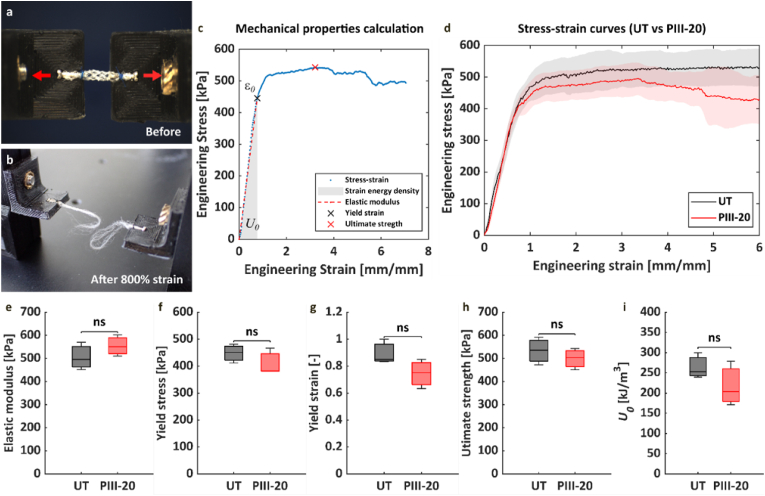
Effect of PIII on tensile properties of MEW tubes (N = 3). Representative images of MEW tubes **a)** before and **b)** after the uniaxial tensile test, with red arrows indicating the tensile direction. **c)** Schematic diagram of the mechanical properties investigated. **d)** Engineered stress-strain curves from the tensile test, with shaded areas representing deviations (± standard deviation). Calculated **e)** elastic modulus, **f)** yield stress, **g)** yield strain, **h)** ultimate tensile strength, and **i)** strain energy density for untreated (UT) and plasma-treated tubes after 20 min (PIII-20). ns: not significant.

Mechanical properties of PIII-treated MEW tubes (PIII-20) were characterized using uniaxial tensile tests and compared to untreated tubes (Fig. 5). A specific attachment was designed to fix and load the tubular scaffolds longitudinally, providing a secure grip on the scaffolds until failure (Fig. 5a and b). The tubular scaffolds were evaluated in terms of different mechanical parameters until failure, as summarized in Fig. 5c. PIII-treated samples exhibited similar stress-strain behavior to untreated samples (Fig. 5d). The elastic modulus of PIII-20 tubes (554.34 ± 45.88 kPa) was comparable to that of untreated tubes (505.90 ± 59.72 kPa), with no statistically significant difference (p = 0.33) (Fig. 5e). The yield strain at which the tubes began to plastically deform was 0.74 ± 0.11 mm/mm for PIII-20 and 0.89 ± 0.09 mm/mm for untreated tubes, respectively (p = 0.14). The yield stresses for PIII-20 and untreated tubes were 410.10 ± 49.12 kPa and 447.90 ± 35.02 kPa, respectively (p = 0.34). The ultimate tensile strengths (UTS), defined as the maximum stress that the MEW tubes can withstand before breaking or delaminating, was 499.12 ± 46.31 kPa for PIII-20 tubes and 533.29 ± 60.16 kPa for untreated tubes (p = 0.48). No differences were observed in terms of strain at UTS (4.14 ± 1.52 vs. 5.90 ± 2.08 mm/mm for PIII-20 and untreated tubes, respectively; p = 0.30). These results indicate that PIII treatment does not have an impact on the mechanical properties of MEW tubes.

The preservation of mechanical properties is attributed to the shallow treatment depth of PIII, which has been reported to generate a nano-thin layer of carbonized structures (typically several tens of nanometers thick), resulting in slight increases in surface stiffness [44]. Such surface-localized changes are minimal relative to the micrometer-scale thickness of MEW fibers. Therefore, the overall bulk mechanical properties of the scaffolds remain unaffected. Furthermore, our preliminary experiments indicated no significant changes in the degradation behavior of PCL scaffolds following the PIII treatment.

### 3.4 Characterization of the optimized PIII-activated MEW tubes

**Fig. 6:**
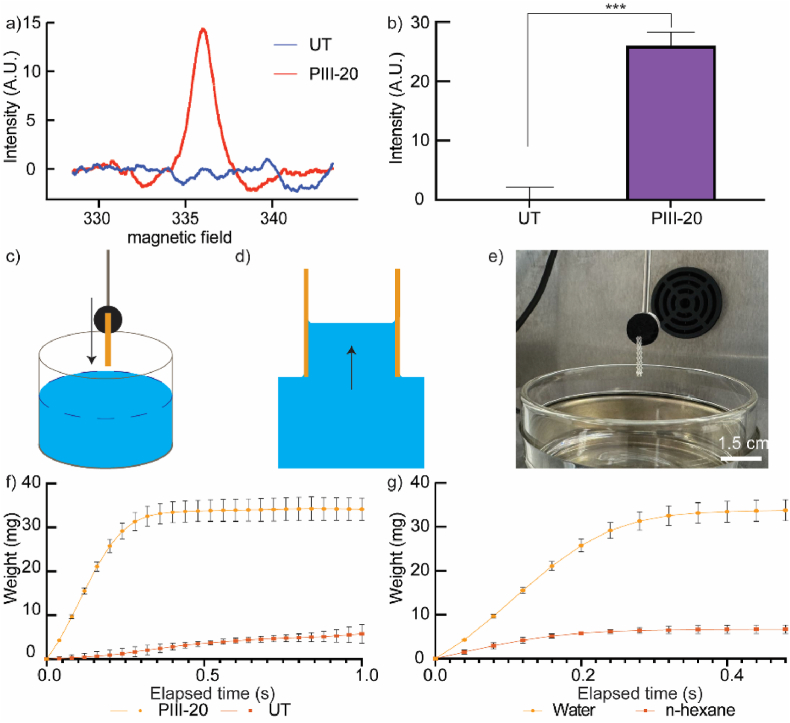
Characterization of the optimal MEW tubes. **a)** Integrated EPR spectra for untreated and PIII MEW tubes within 1 h of treatment, indicating unpaired electrons associated with radicals. **b)** EPR spin density before and after PIII treatment, suggesting the existence of surface embedded radicals. **c)** Schematic representation of wettability measurement set up using **d)** capillary effect. **e)** The optical image of the experimental setup inside the K100 tensiometer. **f)** Water sorption behavior for untreated and PIII MEW tubes. **g)** Sorption behavior of n-hexane compared to water on PIII MEW tubes.

The ability of PIII-treated MEW tubes to stably immobilize biomolecules relies on the presence of surface-embedded radicals [34,35,39].To verify their existence, EPR analysis was conducted on PIII-treated MEW tubes and compared to untreated controls. Unpaired electrons were detected only in the PIII-treated MEW tubes (Fig. 6a), with a significantly higher radical density compared to the untreated control (Fig. 6b), providing direct evidence of embedded radicals. As such radicals are typically stabilized in densified and rich in π-conjugated carbon nanoclusters [60], this observation is consistent with the π-π∗ transition peak observed in XPS analysis (Fig. 3c). Previous studies in our group on various polymeric materials in 2D form suggest that radicals are embedded deeply in the surfaces to depths of several tens of nanometers [39,57,60]. Upon exposure to atmospheric oxygen after removal from the reaction chamber, the radicals can induce surface oxidation, as confirmed by previous XPS and FTIR analyses. Deeply embedded radicals can progressively migrate towards the outermost layer of the surface, facilitating the reagent-free covalent attachment of biomolecules which become in contact with the surfaces [54,60]. The migration can persist for up to a year after PIII treatment, and the embedded radicals can be reactivated by controlling incubation temperature, allowing long-term storage of the samples [40]. Moreover, the radical-based covalent immobilization is generic and has been demonstrated to covalently attach diverse biomolecules on contact [33,56,[61], [62], [63]].

Optimal surface wettability is required for cell-contacting materials, as hydrophilic surfaces maintain the conformation of attached proteins, while hydrophobic surfaces cause their irreversible denaturation. Direct quantification of water contact angles on MEW tubes using a goniometer is challenging due to the absence of a flat surface in the complex structure of MEW tubes [64]. Here, a force tensiometer (Fig. 6c) was used to evaluate the wettability of PIII-treated MEW tubes based on the capillary effect (Fig. 6d)**.** The experimental setup for the wettability measurements was schematically and optically shown in Fig. 6c–e. Fig. 6f shows the sorption behavior of untreated and PIII-treated MEW tubes by measuring the weight of the absorbed liquid as a function of time after being placed vertically against MilliQ water. The untreated control exhibited a sorption rate of 4.6 mg/s, with a maximum absorption of 5.76 mg of water. The low sorption rate and absorbed weight are consistent with the high water contact angle (WCA) of PCL, which has been reported to vary from around 75° to 85° [[65], [66], [67]]. In contrast, PIII-treated tubes demonstrated a significantly higher sorption rate of 132 mg/s and maintained 34.2 mg of absorbed water (Fig. 6f). The increased sorption rate and absorbed water indicate enhanced hydrophilicity of the MEW tubes after PIII treatment. The increase in hydrophilicity can be attributed to the introduction of polar functional groups, such as carbonyl (C=O) groups and free radicals observed in XPS (Fig. 3c), ATR-FTIR (Fig. 4d and e), and EPR analysis (Fig. 6a), which allow for its more favorable interactions with water.

The sorption behavior of water in PIII-treated MEW tubes was compared to that of n-hexane, a total wetting liquid, to estimate their WCA. The sorption rate of PIII-treated tubes against water (132 mg/s) was significantly higher than that against n-hexane (29.4 mg/s), along with a significantly higher absorbed amount (34.2 mg) compared to that of n-hexane (6.70 mg), as shown in Fig. 6g. N-hexane is a commonly used total wetting liquid with a contact angle of approximately 0°. The significantly increased water sorption rate, which was even higher than that of a standard total wetting liquid, suggests that the MEW tube became highly hydrophilic after PIII treatment, consistent with a WCA of 0° on plasma-treated PCL from previous studies [68,69]. The improved wettability is expected to enhance the performance of the porous MEW PCL tubes in the MEW applications, highlighting the potential for maintaining biomolecule conformation after their covalent immobilization.

**Fig. 7:**
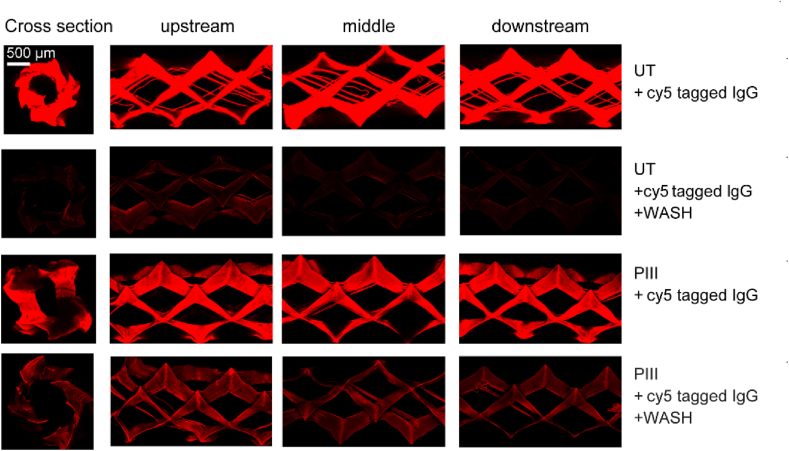
The confocal fluorescence imaging of Cy5-IgG immobilized along the longitudinal cross-section of the untreated and PIII-treated MEW tubes, before and after Tween20 detergent washing. All image acquisition parameters and post-processing procedures were consistent across all presented images.

The ability of PIII-treated MEW tubes to covalently immobilize biomolecules was visualized by fluorescence imaging of the immobilized biomolecules against a detergent wash. Cy5-conjugated immunoglobulin G (Cy5-IgG) was used as a model biomolecule to assess immobilization efficiency. Both untreated and PIII-treated MEW tubes were incubated in a Cy5-conjugated antibody (Cy5-IgG) suspension, rinsed with MilliQ water, and then imaged under confocal microscopy (Fig. 7). Fluorescent Cy5-IgG signals were detected on both untreated and PIII-treated MEW tubes, with higher fluorescence intensity observed on the untreated control, indicating less Cy5-IgG immobilization on the PIII-treated tubes. The reduced Cy5-IgG immobilization on PIII-treated samples can be attributed to their increased hydrophilicity. Hydrophilic surfaces reduce hydrophobic interactions, preventing the conformational change of adhered Cy5-IgG and thereby reducing their aggregation on the material surfaces. The tubes were then subjected to stringent Tween-20 detergent washing to remove all physisorbed Cy5-IgG (Fig. 7). After the Tween-20 wash, Cy5-IgG fluorescence intensity was visibly reduced on the untreated control, indicating that the washing effectively removed the majority of physisorbed Cy5-IgG. In contrast, Cy5-IgG fluorescence signal was homogeneously retained on all surfaces of the PIII-treated MEW tubes, suggesting a homogeneous covalent immobilization of Cy5-IgG to the PIII-treated tubes. As discussed in Section 3.1 and EPR analysis (Fig. 6a), this covalent attachment ability is primarily driven by the radical-based mechanism [39,54]. These results confirm that PIII treatment enables stable and homogeneous covalent immobilization of biomolecules on MEW tubes.

The results demonstrated that the PIII treatment was compatible with the MEW PCL tubes and provided the ability to uniformly immobilize biomolecules across the PIII-treated MEW PCL tube structures. Given that radical-based covalent immobilization is a generic mechanism, as we have previously demonstrated for diverse biomolecules on 2D surfaces [33,[61], [62], [63]], the PIII-treated MEW tubes were subsequently functionalized with VEGF to create bio-instructive surfaces supporting endothelial cell monolayer formation and proliferation, as detailed in the following section.

### 3.5 Immobilized VEGF on PIII-biofunctionalized MEW tubes produced healthy endothelial tissues

The ability to stably and homogeneously covalently attach biomolecules to PIII-treated MEW tubes facilitates the robust immobilization of VEGF, a pro-vascularization growth factor. The MEW tubes were printed with rhombus structures because our previous study showed that this geometry enhanced kidney cell performance [70]. We assessed the *in vitro* effects of the covalently immobilized VEGF on the proliferation and maturation of ciGEnCs, comparing it to traditional VEGF supplementation in cell culture media. CiGEnCs, which model glomerular endothelial cells [56], were used to evaluate the performance of PIII-activated MEW tubes. Untreated tubes in VEGF-supplemented media served as controls, as VEGF is traditionally supplemented in the media. The PIII-treated tubes were functionalized with a range of concentrations of VEGFs (0 ng/mL, 500 ng/mL, 1000 ng/mL), designated to + PIII, +PIII + VEGF500, and +PIII + VEGF1000.

**Fig. 8:**
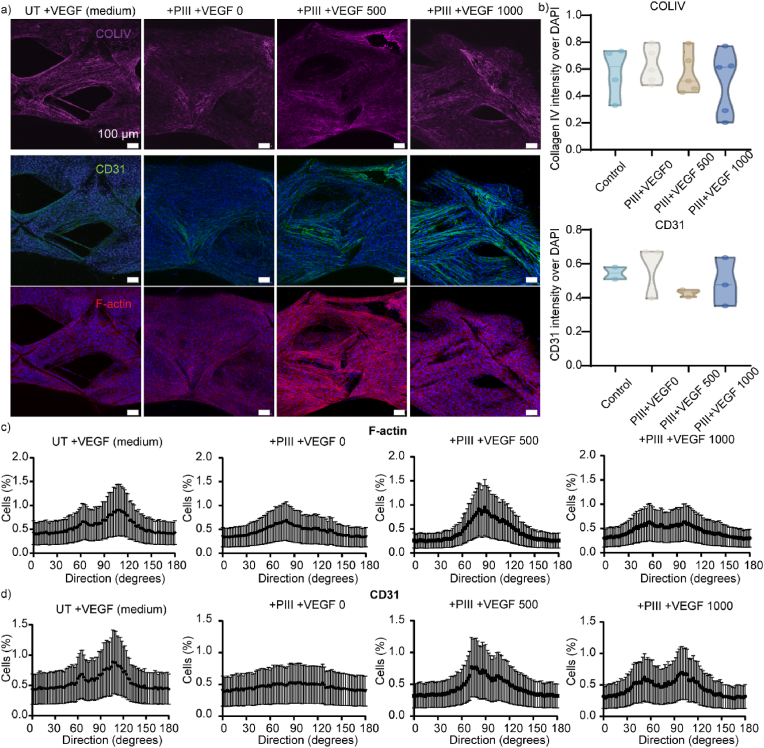
**a)** Immunofluorescent images of ciGEnC cultured in treated MEW tubular scaffolds (collagen IV (magenta), CD31 (green), filamentous actin (red), DAPI (blue)). **b)** Quantification of collagen IV and CD31. **c)** Directionality measurements of filamentous actin for all conditions. N = 3–5. d) Directionality measurements of CD31 for all conditions. N = 3–5.

Representative immunofluorescent images of type-IV collagen (COLIV), a key extracellular matrix component, CD31, a cell-adhesion molecule, and filamentous actin (F-actin), were used to measure cell growth and directionality of ciGEnC on these tubes (Fig. 8a). The amount of COLIV, measured by their fluorescent intensity over DAPI, showed no significant differences across all groups (Fig. 8b). This indicates that the deposition of COLIV on the VEGF-functionalized PIII MEW tubes was similar to the standard control, regardless of VEGF concentration. Additionally, the alignment of ciGEnC was assessed using the directionality measurements of F-actin (Fig. 8c) and CD31 (Fig. 8d). PIII treatment alone resulted in similar F-actin as well as CD31 alignment to all other groups (Fig. 8c and d). The improved cell adhesion observed with PIII treatment could be attributed to the formation of a carbonized layer that can increase wettability and surface oxidation, as observed in previous studies with polymers such as polyurethane, polytetrafluoroethylene, and silk [61,71,72].

Regardless of the VEGF concentration, ciGEnC grown on VEGF-functionalized PIII MEW tubes displayed alignment of both F-actin (Fig. 8c) and CD31 (Fig. 8d) consistent with the suspended VEGF control, with no significant differences. Both VEGF500 and VEGF1000 groups supported the formation of aligned cell layers, indicative of healthy endothelial tissue and formation of microvasculature in the MEW tubes [73]. This demonstrates that covalently immobilized VEGF on PIII-treated scaffolds is effective, similar to traditional methods where VEGF is supplemented and suspended into cell culture media. Importantly, this covalent immobilization method offers a cost-effective alternative, as VEGF remains stable [38,74] and does not require replenishment over the 4-week culture period, unlike the suspension method, where VEGF must be added with every media change. These findings are consistent with our recent study, in which a similar radical-based mechanism was used to immobilize BMP2 on plasma polymer-coated bioceramic scaffolds, retaining the majority of BMP2 on the surface after 45 days of continuous immersion in PBS [75]. This robustness further highlights the efficacy and stability of radical-mediated covalent immobilization.

The established PIII-biofunctionalization process combines the simplicity of physical adsorption with the enduring stability of wet-chemistry covalent immobilization, without inheriting their respective limitations. By creating hydrophilic surfaces, PIII minimizes protein denaturation typically caused by strong hydrophobic interaction in physical adsorption (Fig. 6c–g). The covalent bonds formed between biomolecules and PIII-treated surfaces prevent competition from environmental biomolecules that can desorb and replace physically adsorbed biomolecules [36,37]. Current covalent immobilization methods often apply complex wet-chemistry, such as EDC/NHS and polydopamine, that involves complex chemical steps and may leave toxic residue, limiting scalability in industrial applications as well as hindering its clinical translation and regulatory approval [33]. Requiring only a one-step incubation of the PIII-treated surface in a biomolecule suspension at room temperature for 2 h, the PIII-biofunctionalization process offers a simpler, scalable, and clean solution. Beyond these benefits, our previous studies on 2D surfaces showed greater biomolecule immobilization on PIII-treated surfaces compared to EDC/NHS methods while being detergent-resistant, demonstrating improved covalent attachment effectiveness [76,77]. Therefore, the PIII-biofunctional process provides a superior alternative to physical adsorption and wet-chemistry methods, ensuring that the engineered scaffolds remain effective and practical for tissue engineering applications.

The benefits of the PIII-biofunctionalization process can be extended to other costly growth factors commonly needed for ciGEnC proliferation and maturation, such as human fibroblast growth factors-B and human epidermal growth factors. Since PIII and PIII-related technology can immobilize a variety of biomolecules, including peptides, DNA, and proteins [63,71,78], the PIII-treated MEW tubes could similarly immobilize other bioactive molecules. As demonstrated in this study, these tubes can covalently immobilize VEGF and Cy5-IgG, suggesting potential for future studies to explore immobilizing multiple biomolecules to create a bioinstructive tubular platform while reducing the costs of growth factor supplements. While this study focused on tubular open-pore structures, PIII is not limited to this geometry. For flat scaffolds such as electrospun scaffolds, conventional PIII with a 2D sample holder can be applied [62]. For non-planar 3D-printed porous scaffolds, the treatment can be achieved through different reactor designs, such as packed-bed configurations [38].

A promising future direction for research on the functionalized MEW tubes is to investigate their potential as foundational components in microfluidic device integration, a critical platform for advanced *in vitro* modelling. The combination of MEW and PIII enables the engineering of functionalized tubes that can advance maturation of, e.g., endothelial cells, but could also be used for other tubular structures like kidney tubules.

## 4 Conclusions

Melt electrowriting (MEW) offers unparalleled control over the fabrication of open-pore tubular scaffolds, yet a major challenge remains in biofunctionalizing such structures while maintaining their intricate architecture and mechanical integrity. This study established plasma immersion ion implantation (PIII) as an enabling strategy for biofunctionalizing 3D porous tubular scaffolds fabricated by MEW, achieving homogeneous surface activation while preserving structural integrity and retaining mechanical properties. The PIII-treated surface contained highly carbonized cluster structures that served as a long-term storage of embedded radicals. The PIII-treated surfaces were highly hydrophilic, providing a favorable environment for cell–material interactions. By embedding radicals into the surface of MEW, we demonstrated that PIII facilitates reagent-free covalent biomolecule immobilization in a single step, eliminating the complexity and limitations of wet-chemistry methods.

Building on this new technology, we created a VEGF-functionalized bioinstructive platform, which supports stable endothelialization without requiring soluble VEGF supplementation. The covalently immobilized VEGF effectively promoted the formation and proliferation of an endothelial cell monolayer, yielding results comparable to traditional ciGEnC culture standards while significantly reducing VEGF consumption and associated costs. The vascular structures formed within these MEW tubes hold great potential for integration into bioengineered systems, such as organ-on-chip kidney models, offering a scalable and cost-effective platform for drug screening and disease modeling with reduced reliance on animal testing.

## CRediT authorship contribution statement

**Anyu Zhang:** Writing – original draft, Visualization, Methodology, Investigation, Formal analysis. **Anne Metje van Genderen:** Writing – review & editing, Visualization, Methodology, Investigation, Formal analysis. **Bingyan Liu:** Investigation. **Junyi Qian:** Investigation. **Jirawat Iamsamang:** Investigation. **Ziyu Wang:** Investigation. **Miguel Castilho:** Writing – review & editing, Supervision, Methodology, Investigation, Funding acquisition, Conceptualization. **Behnam Akhavan:** Writing – review & editing, Supervision, Project administration, Funding acquisition, Formal analysis, Conceptualization.

## Declaration of competing interest

The authors declare that they have no known competing financial interests or personal relationships that could have appeared to influence the work reported in this paper.

## Data Availability

Data will be made available on request.
